# Automatic de-identification of French electronic health records: a cost-effective approach exploiting distant supervision and deep learning models

**DOI:** 10.1186/s12911-024-02422-5

**Published:** 2024-02-16

**Authors:** Mohamed El Azzouzi, Gouenou Coatrieux, Reda Bellafqira, Denis Delamarre, Christine Riou, Naima Oubenali, Sandie Cabon, Marc Cuggia, Guillaume Bouzillé

**Affiliations:** 1 Univ Rennes, INSERM, LTSI-UMR 1099, F-35000 Rennes, France; 2 Univ Rennes, CHU Rennes, INSERM, LTSI-UMR 1099, F-35000 Rennes, France; 3 CHU Rennes, Centre de Données Cliniques, Rennes, F-35000 France; 4 IMT Atlantique, INSERM, LATIM - UMR 1101, Brest, F-29238 France

**Keywords:** Clinical de-identification, Distant supervision, Automatic annotation, Named entity recognition, Word representations, Deep learning, French language

## Abstract

**Background:**

Electronic health records (EHRs) contain valuable information for clinical research; however, the sensitive nature of healthcare data presents security and confidentiality challenges. De-identification is therefore essential to protect personal data in EHRs and comply with government regulations. Named entity recognition (NER) methods have been proposed to remove personal identifiers, with deep learning-based models achieving better performance. However, manual annotation of training data is time-consuming and expensive. The aim of this study was to develop an automatic de-identification pipeline for all kinds of clinical documents based on a distant supervised method to significantly reduce the cost of manual annotations and to facilitate the transfer of the de-identification pipeline to other clinical centers.

**Methods:**

We proposed an automated annotation process for French clinical de-identification, exploiting data from the eHOP clinical data warehouse (CDW) of the CHU de Rennes and national knowledge bases, as well as other features. In addition, this paper proposes an assisted data annotation solution using the Prodigy annotation tool. This approach aims to reduce the cost required to create a reference corpus for the evaluation of state-of-the-art NER models. Finally, we evaluated and compared the effectiveness of different NER methods.

**Results:**

A French de-identification dataset was developed in this work, based on EHRs provided by the eHOP CDW at Rennes University Hospital, France. The dataset was rich in terms of personal information, and the distribution of entities was quite similar in the training and test datasets. We evaluated a Bi-LSTM + CRF sequence labeling architecture, combined with Flair + FastText word embeddings, on a test set of manually annotated clinical reports. The model outperformed the other tested models with a significant F1 score of 96,96%, demonstrating the effectiveness of our automatic approach for deidentifying sensitive information.

**Conclusions:**

This study provides an automatic de-identification pipeline for clinical notes, which can facilitate the reuse of EHRs for secondary purposes such as clinical research. Our study highlights the importance of using advanced NLP techniques for effective de-identification, as well as the need for innovative solutions such as distant supervision to overcome the challenge of limited annotated data in the medical domain.

**Supplementary Information:**

The online version contains supplementary material available at 10.1186/s12911-024-02422-5.

## Background

Electronic health records (EHRs) represent a wealth of information that is useful for the advancement of health data reuse for clinical research [1]. However, such healthcare data are extremely privacy-sensitive, as they contain personal identifiable information (PII) about patients and medical practitioners. The use of these EHRs for different secondary purposes represents a real challenge in terms of security and confidentiality [2, 3].

To reuse such records and conduct health data-related studies, the task of de-identification has become essential [4–6]. This is necessary to protect the confidentiality of personal data in EHRs and comply with government regulations set in our case by the French Data Protection Authority, Commission Nationale de l’Informatique et des Libertés—(CNIL),^1^ and the General Data Protection Regulation—(GDPR).^2^

To overcome this problem, several named entity recognition (NER) methods have been proposed to remove or replace such personal identifiers. At first, these techniques were based solely on rules [7, 8], then machine learning and deep learning were studied [4, 9] as well as hybrid systems combining rules and learning [10].

Named entity recognition is an important natural language processing (NLP) task that can be used to extract and classify named entities in text. In the case of sensitive medical data, named entities include personal identifiers such as names, addresses, and phone numbers, as well as other sensitive information. By using the NER to identify named entities in EHRs, records can be automatically deidentified by masking or replacing this information. NER can be used in combination with pseudonymization techniques to identify and replace personal identifiers with pseudonyms, which further enhances the privacy and security of sensitive medical data.

Several recent studies have shown that machine learning-based models trained on annotated datasets achieve better performance than traditional rule-based methods on clinical NER tasks for PII extraction [10]. Dernoncourt et al. [11] proposed a deep learning-based approach based on artificial neural networks (ANNs) for the de-identification of EHRs and presented promising results in two publicly available datasets in English: i2b2 [12] and MIMIC [13]. In France, Paris et al. [9] developed a machine learning model based on a recurrent neural network Bi-directional long short-term memory (Bi-LSTM) associated with a conditional random field (CRF) for the de-identification of hospital reports recorded in an Observational Medical Outcome Partnership (OMOP)^3^ database. Their neural network-based model was trained on a manually annotated set of 2,589 hospital text documents from the Assistance Publique des Hôpitaux de Paris (APHP) and obtained an F1 score of 95.7%. This score was then improved by hybridization with rule-based and knowledge-based methods, achieving an F1 score of 96.7%. Furthermore, for the Italian clinical de-identification scenario [6], the authors have adopted a Bi-LSTM + CRF sequence labeling architecture enhanced by a stacked word representation approach. This method outperforms other state-of-the-art approaches and achieved the best micro-average results on a COVID-19 EHRs dataset.

In such approaches, manual annotation is a crucial step in the process of training NER models, but it also comes with a set of limitations. One of the main limitations is that the process is time consuming and expensive [14], particularly when dealing with large amounts of data and when domain expertise is needed. Furthermore, the limited availability of labeled datasets [15] and the privacy concerns of manual annotation [16] have severely limited the applicability of these supervised techniques in other languages such as French [4]. Moreover, the model trained on a limited amount of data or in a monocentric fashion may not generalize well to unseen data, which could lead to lower performance on new clinical reports or in other hospitals [17]. Additionally, since the annotation is based on a limited dataset, it may not cover all the entities or variations of the entities that the model will encounter in a real-world scenario.

Several studies have accepted the expensive cost of manual annotation and have hired teams to label training data [6]. However, an increasing number of researchers are turning to less expensive techniques to generate automatic labels. One of these techniques is distant supervision, where data are matched with entities in knowledge bases to produce noisy labels [18]. Other approaches include rules for labeling data [9]. Although these techniques are less expensive, they produce noisy labels, which can negatively impact the performance of the resulting model [19]. Approaches such as Snorkel (Ratner et al., 2017) [20] have been proposed, which aim to compensate for the noise of automatic labels by increasing the volume of inexpensive data.

The aim of this study was to develop an automatic de-identification pipeline for all kinds of clinical documents based on a distant supervised method to significantly reduce the cost of manual annotations and to facilitate the transfer of the de-identification pipeline to other clinical centers. The first step was to propose an automated annotation process based on knowledge bases and rules. The second step was to evaluate different state-of-the-art deep learning algorithms to train a NER model for the de-identification task of the following entities: Patient, Doctor, Postal Address, Zip, City, Date, Email, and Phone Numbers.

The feasibility of this study is linked to the availability and use of open data resources, in particular national knowledge bases. In the field of distant supervision, where automatic labels are generated by matching data with entities in knowledge bases, these open data resources proved indispensable. The wealth of information provided by these resources, facilitated the process of automatic annotation, a crucial step in reducing the cost of manual annotation.

## Related works

Automatic de-identification of electronic health records is generally considered a task of named entity recognition, which enables the extraction of personal information from unstructured medical text [5, 21].

Named entity recognition is a core task of natural language processing and a fundamental step in knowledge extraction. The goal is to identify named entities in text and classify them into groups or categories, e.g., person, organization, or date [22].

In recent years, many named entity recognition approaches have been developed and applied to clinical text data, and these techniques can be divided into three broad categories: rule-based systems, machine learning/deep learning-based systems, and hybrid systems combining rules and learning.

### Rule-based named entity recognition

Early works on NER were all founded on rule-based techniques. These NER approaches are systems that consist of developing predefined rules that are elaborated by hand. Rules are based on domain-specific knowledge and lexical features of the targeted entity types.

Several rule-based named entity recognition systems have been developed and extended to the use case of clinical data de-identification [7, 8, 23, 24]**.** In general, rule-based systems provide better performance when annotated data are not available. However, the implementation of these rule-based systems is highly dependent on human expertise in a specific field (clinical domain expertise), which limits their generalization and portability across domains [25].

### Machine learning-based named entity recognition

With the development of machine learning and NLP systems, several methods have been applied to the problem of NER to further automate the process of extracting entities from the text.

Machine learning-based techniques have addressed the named entity recognition problem as a sequence-labeling task. Instead of using rules created by domain experts, machine learning relies on predicting entities in medical text by training models on annotated input examples. Machine learning methods have been successfully used to extract named entities from text with high precision [26].

The most commonly used techniques in the literature include the support vector machine (SVM) [27] and the conditional random field (CRF) [28–30].

Jiang et al. [31] compared two machine-learning approaches, CRF and SVM, for the extraction of clinical entities using a training dataset with 349 annotated notes and a test dataset with 477 annotated notes. In their first experiments on the training set (using a fivefold cross-validation), CRF outperformed SVM with equivalent features. Additional features and kernel optimization for the SVM may improve its performance.

However, it also indicates the complexity of SVM parameter optimization. Based on this comparison, they proposed a novel hybrid clinical NLP system using both ML methods and rule-based components. In the 2010 i2b2/VA NLP challenge, their approach achieved an F1 score of 83,91% for concept extraction and 93,13% for assertion classification.

### Deep learning-based approaches

In recent years, deep learning has significantly improved the performance for several applications in NLP, as well as for NER systems in the medical field. This success of deep learning-based systems for clinical NER results from the combination of two components: contextual embeddings, which are word vectorizations tailored to the context in the text, and high performance of complex neural network architectures.

Currently, several deep learning-based NER models have been implemented on language corpora other than English and have achieved high performance [6, 32–34].

Dernoncourt et al. [11] presented the first deep learning system for the de-identification of patient notes in electronic medical records. They used a recurrent neural network (RNN) model called long short-term memory (LSTM) [35]. They compared the performance of their system with that of CRF-based systems on two de-identification datasets: i2b2 [8] and MIMIC [13]. It achieved an F1 score of 97.85% on the 2014 i2b2 de-identification challenge dataset and an F1 score of 99.23% on the MIMIC de-identification dataset.

These RNN-based architectures have been further improved. In their paper, Huang et al. [36] performed a comparative study of several LSTM-based models for sequence tagging. These models include LSTM networks, Bi-LSTM networks (Bi-LSTM), LSTM with a conditional random field layer (LSTM-CRF), and bidirectional LSTM with a CRF layer (Bi-LSTM—CRF). The results demonstrate that the hybrid Bi-LSTM—CRF model outperforms the other models. It achieved an F1 score of 94.40% on the CoNLL2000 dataset and an F1 score of 84.74 on the CoNLL2003 dataset.

For the de-identification task, Liu et al. [37] proposed a hybrid system that combines the Bi-LSTM—CRF architecture with a rule-based subsystem. The results of the proposed model on the i2b2 dataset achieve F1 scores of 96.98%, 95.11% and 98.28% under the "token", "strict" and "binary token" criteria, respectively, thus outperforming the results of Dernoncourt et al. [11] on the same de-identification dataset. The hybrid Bi-LSTM—CRF architecture for sequence labeling was then improved by adding contextualized word embeddings (BERT embeddings) [38], attaining remarkable F1 scores on the i2b2 2014 dataset. Specifically, it achieved scores of 97.48%, 95.50%, and 98.70% under the "token," "strict," and "binary token" criteria, respectively.

Large pretrained models, such as BERT (Bidirectional Encoder Representations from Transformers) [39], CamemBERT [40] language model for French and FlauBERT [41] have rapidly become the state-of-the-art approach to model tasks in NLP. For the NER task, these large pretrained models are typically used in two different ways: the first uses the transformers to provide contextual word embeddings for a standard Bi-LSTM—CRF sequence labeling architecture, and the second fine-tunes the transformers on an NER task with the addition of a linear layer for word-level predictions [42]. These findings have been applied to clinical NER [43] and then to the de-identification of medical records in France [4].

All these approaches have one limitation, deep learning models trained on sensitive data are not sharable. Moreover, they are difficult to train locally, as they require numerous annotations. It is therefore important to consider a method for reducing the cost and duration of annotations.

### Word representations & BiLSTM-CRF architecture

#### Word representation

The representation of tokens in the text is an essential part of many NLP tasks, including clinical NER. Traditional word embeddings, such as Global vectors for word representation GloVe [44] and Word2Vec [45] provide only one global representation for each word in the text. However, words can have different meanings depending on their context. Contextual embeddings address this limitation by providing a representation for each word based on its context, allowing for the capture of many syntactic and semantic properties of words in various contexts.

We present the following methods currently used in the literature to generate contextual embeddings.

##### BERT pretrained language model

Proposed by Devlin et al. (2018) [39], BERT, which stands for bidirectional encoder representations from transformers, is a pretrained language model for text representation based on the transformer architecture. The representation made by BERT has the particularity of being contextual. Moreover, the BERT context is bidirectional; that is, the representation of a word involves both the words that precede it and the words that follow it in a sentence. In this work, we used the mBert model: BERT multilingual base model (cased), a multilingual BERT pretrained on the top 104 languages with the largest Wikipedia.

##### CamemBERT

Developed by Facebook, INRIA, and Sorbonne University, CamemBERT [40] is a state-of-the-art language model for French based on the RoBERTa architecture [46], which is a variant of BERT. Pretrained on large French corpora, this model has been evaluated on four NLP tasks: part-of-speech tagging (POS), dependency parsing, named entity recognition (NER), and natural language inference. CamemBERT improved the state of the art in the four previous tasks, confirming the effectiveness of large pretrained linguistic models for French [40].

##### FlauBERT

FlauBERT, French language understanding via Bidirectional Encoder Representations from Transformers, is a French language model [41]. FlauBERT has been trained on a very large and heterogeneous French corpus, with a configuration similar to BERT and CamemBERT. This model was evaluated on several NLP tasks, and the results demonstrate yet again that a French-language model gives better performance than multilingual BERT models as well as other French language models [41].

##### FLAIR

Proposed by Akbik et al. (2018) [47], Flair embeddings are contextual string embeddings. They are generated from a character language model that is trained by predicting the next character based on previous characters. Flair embeddings model words as sequences of characters, which allows for a better representation of misspelled words often present in medical reports. In addition, these types of embeddings are contextualized, so the same word will have different embeddings due to different context usage.

##### FastText

FastText is a fast word representation technique proposed by Bojanowski et al. (2017) [48]**.** Trained on large corpora, this approach is based on the skipgram model, where each token of the text is represented by a sequence of n characters and n grams. For example, for *n* = 3 (trigram), the word "Patient" will be represented by < Pa, Pat, ati, tie, ien, ent, nt > , where ' < ' and ' > ' represent the beginning and end of the word. This n-gram information enriches word vectors with subword information and allows morphological information to be captured to construct vectors for unseen words or out-of-vocabulary words.

#### Bi-LSTM

Bidirectional long short-term memory (Bi-LSTM) is a type of recurrent neural network (RNN) that extends the capabilities of traditional Long Short-Term Memory networks (LSTMs). First introduced by Hochreiter and Schmidhuber in 1997 [35], LSTMs are a variant of RNNs widely used in the literature and highly effective in addressing the challenge of learning long-term dependencies in sequential data.

In the context of natural language processing, Bi-LSTMs are designed to model the contextual information of each word, as they are used to capture past and future information [49]. Unlike traditional LSTMs, which process input sequentially, Bi-LSTMs process the sequence in both forward and backward directions, merging information from both directions.

#### CRF

Conditional random fields (CRFs) are a framework used to construct probabilistic models to partition and label sequential data [50]. They offer a unique combination of properties: discriminatively trained models for sequence segmentation and labeling.

This framework was proposed by Lafferty et al. (2001) [50], and its use is constantly growing. For instance, the CRF model is the most widely deployed in NER tasks and especially in de-identification tasks due to both its theoretical advantage and its experimental efficiency [37].

The purpose of using CRF as the last layer is to ensure that the label produced by Bi-LSTM is valid by learning the adjacent relationship between the labels, as LSTM can only consider the long-term context information of sentences; thus, it cannot consider the dependencies between labels [33]. This makes the CRF an advantageous option for decoding.

## Methods

In this section, we present an overview of the materials and methods used in this study.

**Fig. 1:**
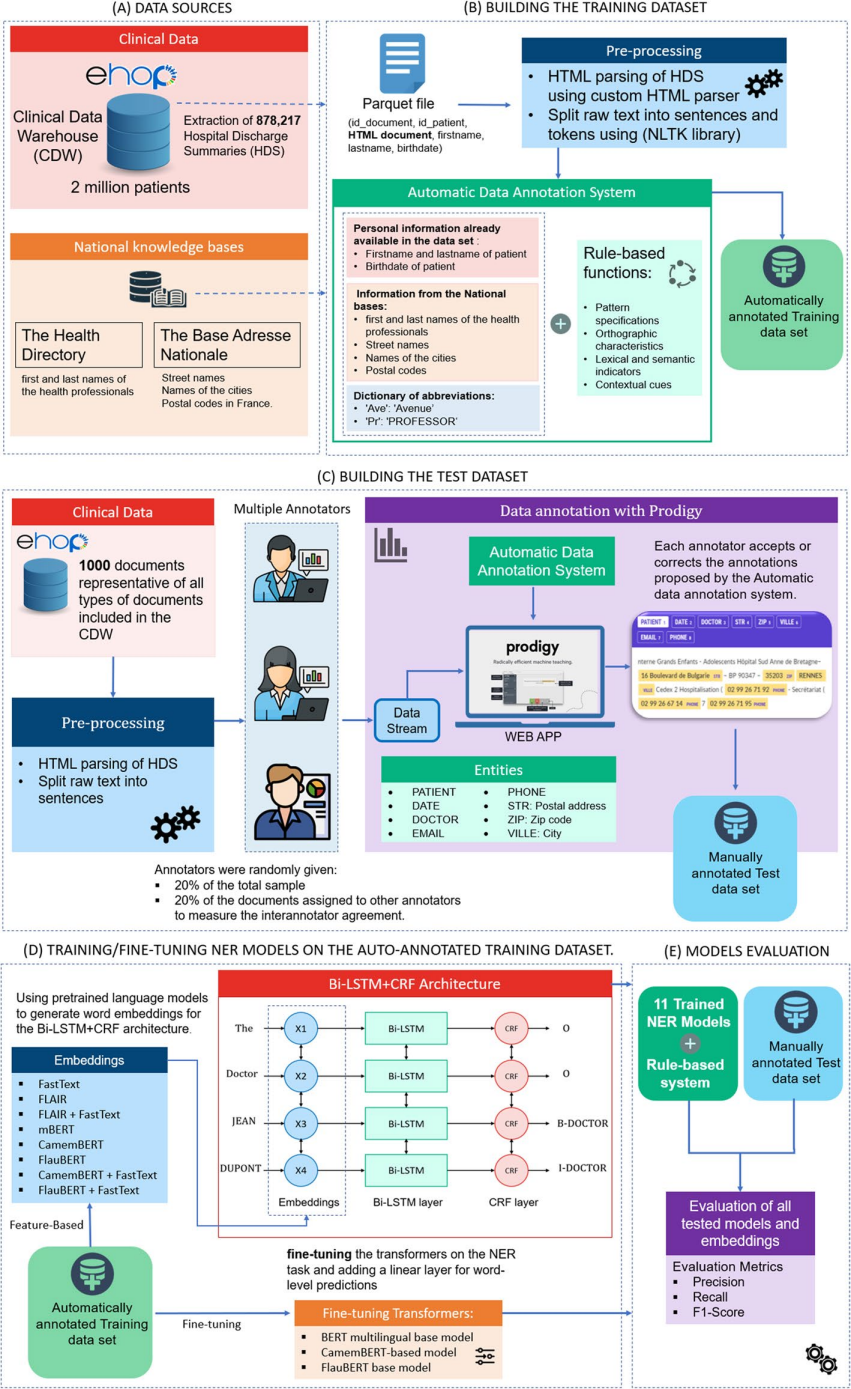
Overview of the de-identification process

Figure 1 illustrates the process of de-identification of clinical notes in French.

A full description of the datasets, knowledge bases, preprocessing techniques and automatic annotation procedure used to create the training dataset is presented. In addition, the NER methods used in this study and the experimental parameters are clarified. Additionally, the manual annotation procedure used to create the test dataset for evaluating the trained models is described. Finally, statistics of the de-identification dataset and inter-annotator agreement are presented at the end of this section.

## Data sources

The databases used in our study consist of clinical data from the eHOP Clinical Data Warehouse (CDW) at Rennes University Hospital in France [51] and knowledge bases including national knowledge bases of French streets and city names from the Base Adresse Nationale (BAN) [52] and healthcare professionals practicing in France from the Health Directory [53].

Our method exploits both the clinical data from the CDW and the public identification information from the knowledge bases as well as author characteristics to perform automatic data annotation.

### Clinical data

We used the eHOP Clinical Data Warehouse of the Rennes University Hospital, France, to retrieve the EHRs of patients [51]. The CDW allows us to retrieve both structured and unstructured data from approximately 2 million patients who came to the hospital since 2000. The documents come from clinical applications in either native HTML, CDA R2, XML or pdf formats and are then transformed to HTML. Each document is associated with a given venue of a given patient. Hence, several metadata can be retrieved to contextualize documents such as the type of document coded in LOINC terminology, first name, last name, birthdate, address, phone, and mail of the patient, and the date of stay.

### Knowledge bases

We collected data from national open sources knowledge databases of French streets, city names, and health professionals practicing in France [52, 53].

The Health Directory gathers identification data for healthcare professionals and their structures from various national repositories, including the Shared Directory of Professionals involved in the healthcare system (RPPS), Association for the Development of Computer Logic (ADELI), and National File of Healthcare and Social Institutions (FINESS). These data are classified into two categories: data accessible to all and restricted access data. The freely accessible data, for which extractions are published, include the following:

- The RPPS or ADELI number.
- Gender.
- Full name of professional.
- Professional category: civilian, military, student.
- Profession.
- Mode of practice: private practice, employed, volunteer.
- Qualifications: degrees, practice authorizations, skills.
- Contact information for the practice structures.
- Function and type of activity.

The Base Adresse Nationale (BAN) is one of the nine databases in the public service for reference data. It is the only officially recognized address database by the administration. The BAN is accessible in the form of files and APIs and includes the following data:

- Street name (nom_voie).
- Postal code (code_postal).
- INSEE code.
- City name (nom_commune).
- Old municipality INSEE code.
- Old municipality name.
- Longitude (lon).
- Latitude (lat).
- Position type.
- Source street name (source_nom_voie)
- Municipality certification (certification_commune)
- …

From these resources, we selected valuable data for our study, such as the first and last names of the health professionals (“Nom d’exercice” and “Prenom d’exercice”), the street names (“nom_voie”), the names of the cities (“nom_commune”), and the postal codes (“code_postal”) in France.

## Building the training dataset

We extracted 878,217 hospital discharge summaries (HDSs) from the CDW. The data are then stored in Parquet files on a local HDFS node with metadata (id_document, id_patient, HTML document, firstname, lastname, birthdate).

### Data preprocessing

We first parsed HTML discharge summaries using a custom HTML parser based on the Python “*html.parser*” library. This parser allows tracking character positions of extracted raw texts in the original HTML documents so that we can mask potential PII at the end of the pipeline. Once the HTML is parsed, raw text is split into sentences and tokens using the NLTK library^4^. This module allows us to split paragraphs into sentences according to punctuation and capitalization. At the end of the preprocessing steps, each token can be positioned in its original position in the HTML document.

### Data annotation

**Fig. 2:**
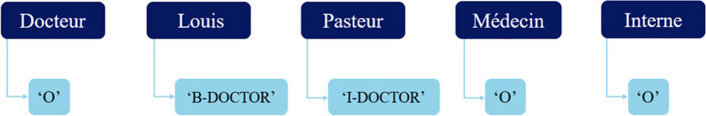
BIO tagging format

In this article, we adopted the BIO formatting scheme [54], where a token is labeled as a ‘B-tag’ if the token is in the beginning of a named entity, or an ‘I-tag’ if the token is inside a named entity, otherwise an ‘O’ for ‘Outside’. An example of BIO tagging is shown in Fig. 2.

We performed an automatic annotation for the following eight entities:

- PATIENT: Last name and first name of the patient
- DATE: All dates mentioned, including date of birth, date of admission to the hospital, and date of discharge
- DOCTOR: Last name and first name of health professionals
- EMAIL: Email addresses
- PHONE: Phone and fax numbers
- STR: Postal address, designation of a location
- ZIP: Zip code
- VILLE: Name of the city

Dictionary-based markers were used for the DOCTOR, STR, ZIP, and VILLE entity types. We performed data normalization and data augmentation by splitting the names of the compound health professionals into several words, for example, “JEAN-PIERRE” gives ('JEAN-PIERRE', ‘JEAN’, and ‘PIERRE’). We filtered the resulting dictionaries to exclude ambiguous terms such as complementary pronouns (*e.g.,* ‘DE', 'DU', 'DES', 'LE', 'LA', 'L', 'D', etc.). Finally, we added a dictionary of abbreviations for street names and academic titles of health professionals, for example: 'Ave': 'Avenue', 'St': 'Saint', 'Dr': 'DOCTOR', 'Pr': 'PROFESSOR', etc.

The rule-based system includes a set of rules that exploit numerous components, including personal information already available in the dataset (*e.g.,* patient names and their dates of birth) and the results of the dictionaries, to recognize entities in the text. For each type of entity, we developed a Python function that combines the previous components with several characteristics:

- Pattern specifications, which incorporate the classic lexical forms of certain types of entities appearing in medical reports, for instance dates (*e.g.,* dd-mm-yyyyy, dd/mm/yy), zip codes (00000), and phone numbers (*e.g.,* ‘00–00-00–00-00–00,’00 00 00 00’, ‘00.00.00’).
- Orthographic characteristics, which consist of word specifications such as words that are capitalized, words that begin with upper or lower case, and the length of tokens or words.
- Lexical and semantic indicators. As an example, street names often include terms such as "AVENUE", "BOULEVARD", "RUE”, and “ALLEE”.
- Contextual cues that point to the presence of a particular type of entity in the clinical text. They include specific lexical expressions (*e.g.,* titles of healthcare practitioners, months/days and their abbreviations, common abbreviations used in French medical reports, etc.

Finally, we selected only the sentences that contain at least one entity, and we divided the documents in such a way that for each patient, all his documents go to the same dataset (*Train_auto:* 80%, *Valid_auto:* 10%, and *Test_auto:* 10%) using the Permanent Patient Identifier (*ID_PAT*).

## System architecture

We formulate the medical de-identification problem as a sequence labeling task. For example, given an input sentence "Monsieur le Docteur JEAN DUPONT ", the medical de-identification model will generate the following labeling sequence "[O] [O] [B-DOCTOR] [I-DOCTOR]".

We have used two different approaches for NER based on the pretrained language models currently used in the literature.

In the first one, we used language models to provide embeddings to one of the best architectures for sequence labeling proposed in the literature by Huang Z et al. [36]. This architecture is based on the Bi-LSTM—CRF model. In general, the architecture consists of three layers: (1) a word representation layer, (2) a Bi-LSTM layer, and (3) a CRF layer.

The input embedding layer converts each word of a sentence into a sequence of vector representations. This sequence is then input into the Bi-LSTM layer, which outputs a sequence of vectors containing the probabilities of each label for each corresponding token. Finally, the CRF layer uses these probability vectors to predict the most likely sequence of labels.

We used embeddings from language models like Multilingual BERT (mBERT), CamemBERT, and FlauBERT as well as other embeddings like Flair and FastText adapted to the French language used in EHRs.

In the second, we fine-tune the language models themselves on the NER task and add only a linear layer for word-level predictions [42].

## Experimental settings

In this subsection, we present all the combinations of models and pretrained language models for text representation that we have used for our study. Model hyperparameters were selected from the literature and constrained by GPU memory allocation. We trained each model independently on the automatically annotated training set and then tested it on the manually annotated test set.

### Computing resources

All experiments were performed on a secure server hosted at the University Hospital of Rennes with 112 CPU cores: Intel(R) Xeon(R) Gold 6258R and an NVIDIA A100 40 GB graphics card.

### Training parameters

#### Bi-LSTM—CRF Based Models

We used the BERT multilingual base model (cased) (mBERT), which contains 12 layers, 768 hidden dimensions, 12 attention heads, and 179 M parameters. In addition, we used the PyTorch Deep Learning Framework to implement the mBERT + Bi-LSTM + CRF model. The model was trained for 5 epochs using the Adam optimizer. Table 1 lists the hyperparameters used to train the model.

**Table 1:** Hyperparameters of the BERT-based model

Hyperparameter	Value
Attention heads	12
Batch size	64
Epochs	5
Hidden size	768
Hidden layers	12
Maximum Sequence Length	512
Parameters	179 M
Optimizer	Adam

To further explore the performance of our models, we used the Flair11 library [55] for the implementation of the Bi-LSTM and CRF model with the embeddings mentioned earlier. FLAIR is a natural language processing (NLP) framework built on the popular PyTorch deep learning library. FLAIR enables state-of-the-art performance and includes implemented architectures such as Bi-LSTM and CRF as well as very powerful embeddings such as Flair embeddings, FastText embeddings, BERT embeddings, etc. We used the French FastText embeddings alone, then the French Flair embeddings (forward and backward), and finally, we combined both Flair and FastText embeddings using the StackedEmbeddings () function of FLAIR. We used the Bi-LSTM and CRF model with these embeddings. The hyperparameters used in this experiment are reported in Table 2.

**Table 2:** Hyperparameters of the FLAIR + FastTEXT-based model

Hyperparameter	Value
Hidden size	256
Batch size	512
Learning rate	0.1
Max_epochs	5
Locked_dropout	0.5
Word_dropout	0.05
Patience	3
Anneal_factor	0.5
RNN layers	1
Optimizer	Stochastic gradient descent (SGD)

We also used the CamemBERT-based model, pretrained on a subcorpus of the OSCAR multilingual corpus [56]. The CamemBERT embeddings + Bi-LSTM + CRF model was trained for 5 epochs using the Adam optimizer. The maximum sequence length is set to 512, the batch size is set to 64, the learning rate is set to 5e-5 and the dropout is set to 0.5.

Following the experiments conducted by Suárez PJO et al. [43], we also tested the combination of CamemBERT embeddings and FastText embeddings with the Bi-LSTM + CRF architecture.

Finally, we applied the FlauBERT base-cased model available on the Hugging Face library,^5^ which contains 12 layers, 768 hidden dimensions, 12 attention heads, and 138 M parameters. We then ran the FlauBERT embeddings + Bi-LSTM + CRF and FastText + FlauBERT embeddings + Bi-LSTM + CRF models on the 4,948,186 training data with the same hyperparameters as CamemBERT.

#### Fine-tuning transformers

We used pretrained language models to generate word embeddings for the Bi-LSTM + CRF architecture. In addition, we tested fine-tuning the BERT model and other French versions of BERT, which is a common approach for various NLP tasks. This involved fine-tuning the transformers on the NER task and adding a linear layer for word-level predictions [42]. The models we used included the BERT multilingual base model, the CamemBERT-based model, and the FlauBERT base model.

We used the Hugging Face Transformers Framework [57] to fine-tune the models on the training sentences. The models were trained for 5 epochs using the Adam optimizer, the batch size was set to 16, and the learning rate was set to 5e-5.

## Evaluation

### Building the test dataset

To evaluate the quality of our automatic annotation system, we manually annotated a set of documents representative of all types of documents included in the CDW. Several types of documents were excluded because they did not mention identifying data, such as Diagnosis-related groups (DRGs) and drug administrations.

Data sampling consisted of a random selection of 250 documents stratified in each original data format (that is, native HTML, CDA R2, XML, PDF). Therefore, the total evaluation dataset to be annotated includes a total of 1000 documents. The details of the distribution of document types in the sample are available in (Additional file 1).

Annotators were selected among the members of the Massive Data and Learning Information Systems in Health (DOMASIA) team of the Signal and Image Processing Laboratory (LTSI) and signed a confidentiality agreement. Five people with different backgrounds performed the annotation: three medical doctors in public health with medical informatics backgrounds, one data manager, one PhD student in NLP, and one Master’s student in NLP.

Each annotator was randomly assigned 20% of the total sample (n1), that is, approximately 200 documents per annotator. Then, to evaluate the concordance and to measure the inter-annotator agreement, each annotator also annotated 20% of the documents assigned to each of the other annotators (n2).

In total, each annotator annotated the following number of documents presented in Table 3.

**Table 3:** Total number of documents annotated by each annotator

Annotator	n1	n2	Total
1	187	140	327
2	195	171	366
3	226	177	403
4	192	151	343
5	200	161	361
6	185	148	333

After distributing the documents among the annotators, each document went through a pre-processing phase, which included parsing HTML documents using a customized HTML parser and splitting the raw text into sentences. This pre-processed data was stored in a JSONL file. In general, Prodigy prefers line-break delimited JSON, as it can contain detailed information and metadata, and can be read line by line. An example of a JSONL file entry is provided for clarity:

- *{"text": "CENTRE HOSPITALIER UNIVERSITAIRE DE RENNES …", "meta": {"ID_PAT": 363342.0, "ID_ENTREPOT": 141303567.0, "CODE": "EHOP:CR_ECHO", "annotator": 5.0, "annotator2": 2.0}, "tokens_parser": [{"start_char": 1713, "end_char": 1719, "text": "CENTRE", "start": 0, "end": 6, "id": 0}, {"start_char": 1720, "end_char": 1738, "text": "HOSPITALIER", "start": 7, "end": 18, "id": 1}, {"start_char": 1732, "end_char": 1764, "text": "UNIVERSITAIRE", "start": 19, "end": 32, "id": 2}, {"start_char": 1746, "end_char": 1781, "text": "DE", "start": 33, "end": 35, "id": 3}, {"start_char": 1749, "end_char": 1791, "text": "RENNES", "start": 36, "end": 42, "id": 4}]}.*

The "*text*" field contains the content of the sentence. The "*meta*" field contains metadata, including the patient identifier ("ID_PAT"), the warehouse identifier ("ID_ENTREPOT"), a specific code ("CODE") and the annotator identifiers ("annotator" and "annotator2"). The "tokens_parser" field provides the character positions of extracted raw texts in the original HTML, specifying start and end positions, the text of each token and its ID.

The data (Jsonl files) selected for the annotation campaign were collected on a secure local server.

Manual annotation was performed with the Prodigy annotation tool at the sentence level. The guidelines for annotations were shared with all annotators through a dedicated document. A demonstration was also performed on a test sample so that the annotators could become more familiar with the Prodigy [58] annotation tool.

**Fig. 3:**
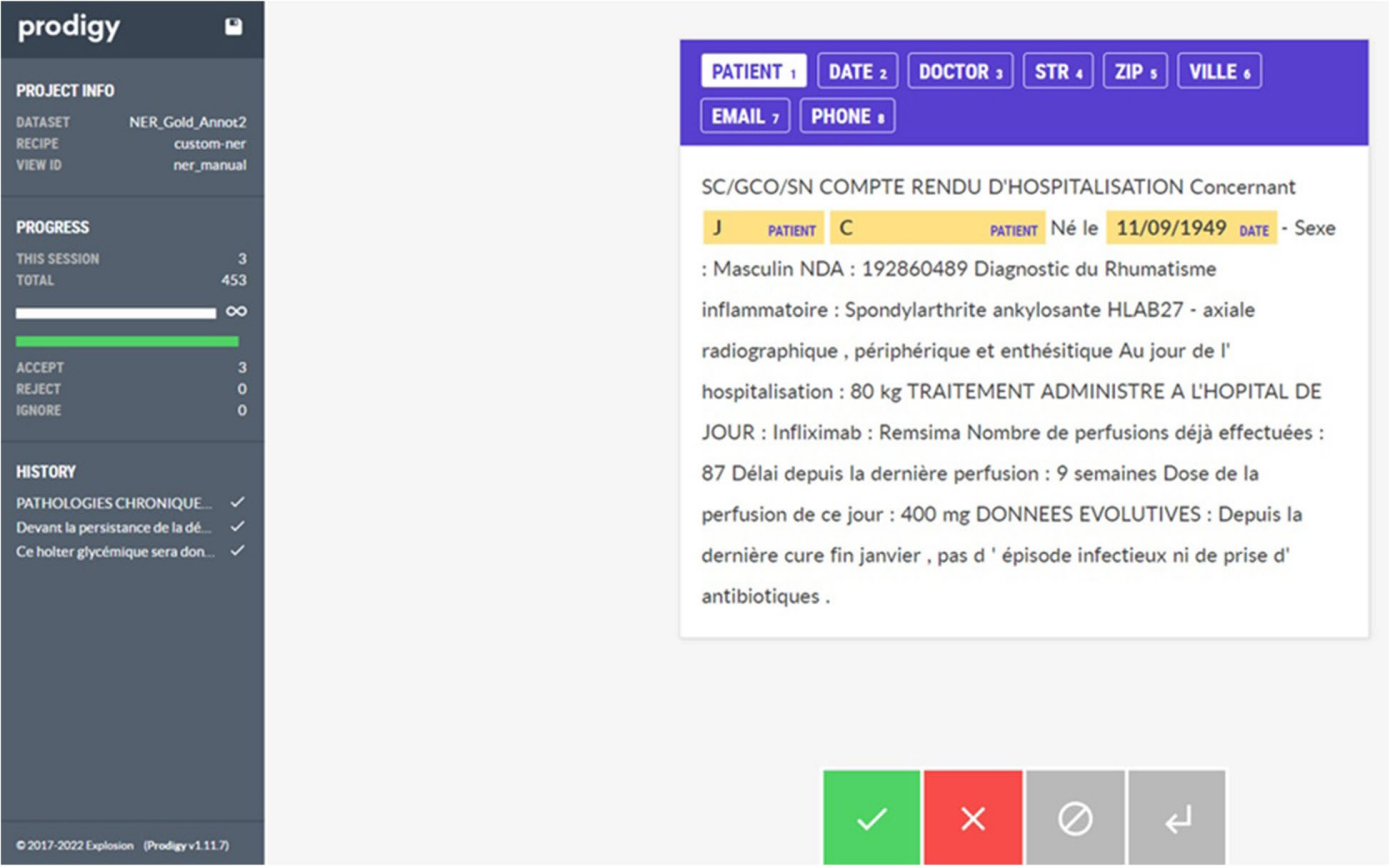
Annotator UI for manual annotation

During manual annotation, each annotator examined all the sentences assigned to them. The pre-annotation step, implemented using a rule- and knowledge-based system, aimed to simplify the annotation task by highlighting the detected entities in the Prodigy interface. In some cases, the rule- and knowledge-based system did not detect any named entity in the sentence, and the annotators corrected this by annotating the entities present in the sentence. This method enabled us to simplify the manual annotation task by continuously processing the sentences and highlighting the detected entities based on rules and dictionaries, making it convenient for human annotators to simply accept, reject, or correct the annotations. An image of the user interface is shown in Fig. 3. No difference was observed between annotating raw sentences or pre-annotated sentences on inter-annotator agreement or model performances (data not shown).

### Evaluation metrics

There are several evaluation metrics commonly used to evaluate the performance of named entity recognition (NER) systems. In this work, we selected three metrics: precision, recall, and F1 score.

Precision is the number of correctly predicted entities (true positives) divided by the total number of predicted entities. Measures how many of the entities predicted by the model are correct. Recall the number of true positives divided by the total number of actual entities. Measure how many of the actual entities were correctly predicted by the model. The F1 score is the harmonic mean of precision and recall and is often used as a single metric to evaluate the overall performance of an NER system. It considers both the precision and recall of the model and is a good overall measure of performance.

The metric equations are shown in Table 4, where TP (true positive) is the number of entities that the model can correctly predict. FP (false positive) is the number of irrelevant entities that the model recognizes. FN (false negative) is the number of correct entities that the model does not predict.

**Table 4:** Evaluation Metrics Equations for NER

Metric	Equation
Precision	${\text{P}}={\text{TP}}/({\text{TP}}+{\text{FP}})$
Recall	${\text{R}}={\text{TP}}/({\text{TP}}+{\text{FN}})$
F1-Score	${\text{F}}1=2 *{\text{P}}*{\text{R}}/({\text{P}}+{\text{R}})$

## Statistics of the de-identification dataset

**Fig. 4:**
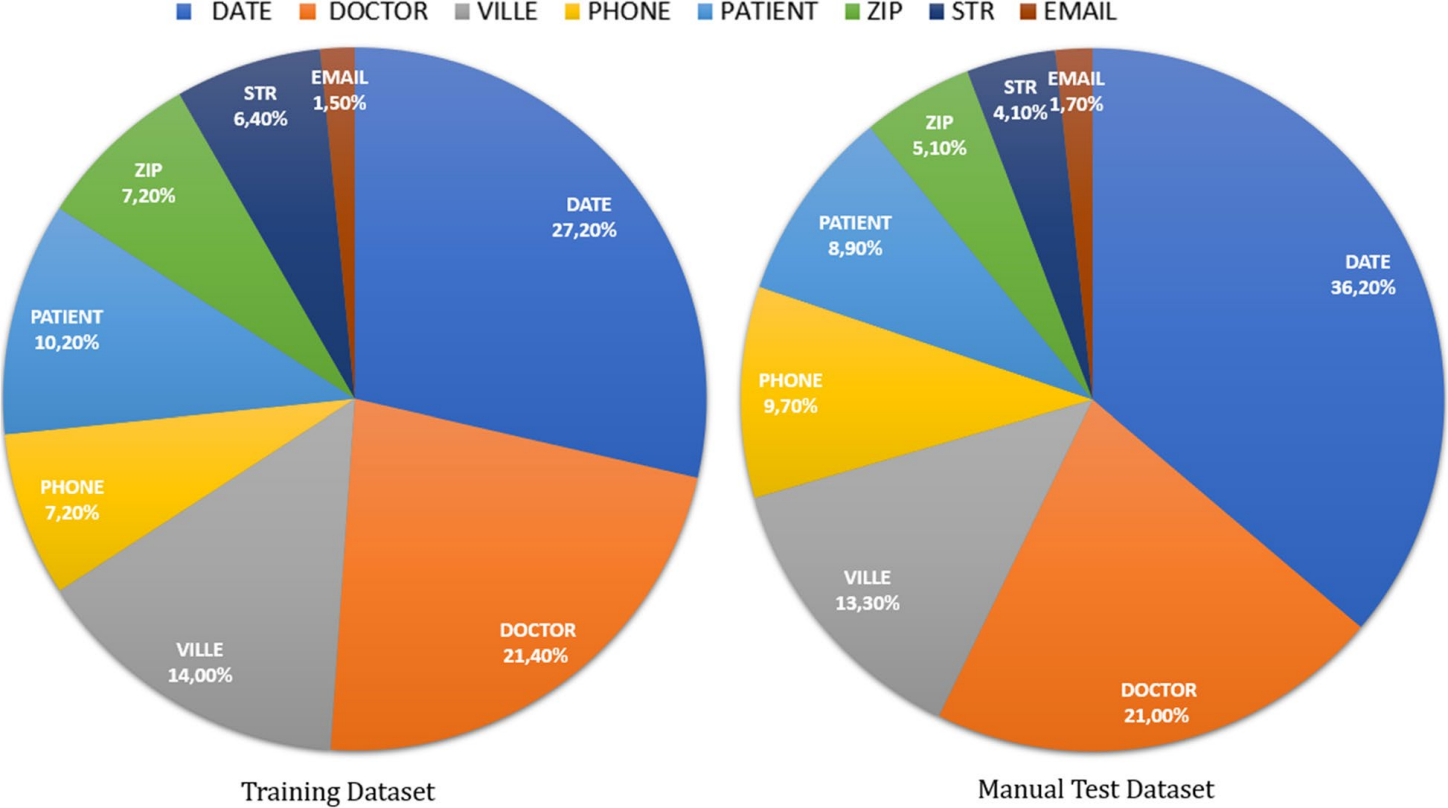
PII Entity distribution in the training and manual test dataset

Our data source contains more than 900 K EHRs. We split the documents into sentences and filtered out sentences that do not contain any of our targeted entities in the automatic annotated dataset. The dataset is rich in terms of PII instances belonging to our eight classes. Some statistical data on the de-identification dataset are reported in Table 5. In addition, the frequency of entities in the training and manual test datasets is shown in Fig. 4.

**Table 5:** Statistical data concerning the de-identification dataset

Data split/annotation method	#Sentences	#DOCTOR	#PATIENT	#DATE	#VILLE	#ZIP	#STR	#EMAIL	#PHONE
training/automatic	4,948,186	3,883,360	1,853,646	4,948,519	2,544,287	1,305,402	1,165,009	276,208	2,210,577
validation/automatic	608,305	479,821	229,925	607,383	315,771	162,791	144,654	35,288	271,081
test/automatic	620,581	489,008	232,030	620,028	322,279	165,492	147,432	35,322	276,873
**test/manual**	**23,196**	**1206**	**510**	**2078**	**764**	**293**	**234**	**96**	**545**

Table 5 provides the number of sentences that contain at least one of the target entities and number of mentions for each entity class for different splits of the de-identification dataset.

The entity distribution analysis reveals quite similar patterns in the training and manual test datasets. In both datasets, DATE entities are quite frequent, representing 27.2% and 36.2% respectively. This high frequency underlines the importance of dates in EHRs. Another important similarity is observed in the distribution of DOCTOR entities. In the training dataset, they represent 21.4%, and in the test dataset, 21.0%. This consistent representation of DOCTOR entities indicates the significant presence of health professional mentions in both datasets.

This uniform distribution also extends to other entity classes such as VILLE, PHONE, PATIENT, ZIP, STR and EMAIL, for which the distribution percentages remain consistent between the two datasets.

## Inter-annotator agreement

The concordance on the annotation task was evaluated on a set of 51 documents corresponding to 51 different patients. In total, there were 2,647 sentences and 20,570 tokens. The 51 documents belonged to 27 different types of medical documents. Examples of document types include "*Emergency Admission*", "*Surgical Reports*", "*Administrative*", "*Letters to the Patient*", and more. The goal is to reflect the consistency of annotations between different annotators for each specific category of medical document.

The Fleiss kappa coefficient was used to measure the overall inter-annotator agreement. A value of 0.93 (95% CI: 0.922–0.939) was obtained, indicating a very good level of agreement. In addition, the concordance was evaluated for each pair of annotators as well as for each label and each document type. The results of this analysis are presented in Table 6 and 7, and the detailed annotator concordances by type of document are available in (Additional file 2). The results showed a high level of concordance for all types of tags and documents, with concordance measures ranging from 0.87 to 1. The concordance was highest for the PHONE and EMAIL tags, while the lowest concordance was for the PATIENT tag. Regarding the document types, the annotations showed the highest concordance for emergency reports, operative reports, administrative documents, and letters to patients.

**Table 6:** Agreement between annotators

	**Annotator 1**	**Annotator 2**	**Annotator 3**	**Annotator 4**	**Annotator 5**	**Annotator 6**
Annotator 1	1 (1,1)	NA	NA	0.909 (0.861,0.956)	0.880 (0.843,0.916)	0.97 (0.952,0.987)
Annotator 2		1 (1,1)	0.874 (0.784,0.965)	0.965 (0.942,0.988)	0.893 (0.868,0.917)	0.962 (0.945,0.978)
Annotator 3			1 (1,1)	0.986 (0.968,1)	0.928 (0.905,0.952)	0.943 (0.908,0.978)
Annotator 4				1 (1,1)	0.861 (0.817,0.904)	0.961 (0.937,0.985)
Annotator 5					1 (1,1)	0.91 (0.871,0.949)
Annotator 6						1 (1,1)

**Table 7:** Agreement between annotators for each tag

Tag	Fleiss
O	0.93 (0.922,0.939)
STR	0.96 (0.945,0.976)
ZIP	0.895 (0.847,0.942)
VILLE	0.947 (0.922,0.973)
PATIENT	0.887 (0.852,0.922)
DATE	0.948 (0.929,0.967)
DOCTOR	0.949 (0.935,0.963)
PHONE	0.992 (0.986,0.998)
EMAIL	1 (1,1)

## Results

In this section, we present the results of our study, including the evaluation results of all tested models on the manually annotated test dataset, the performance per entity class of the best model and the rule-based approach and the detailed performance results of our best model for different types of medical documents.

## Evaluation results

**Fig. 5:**
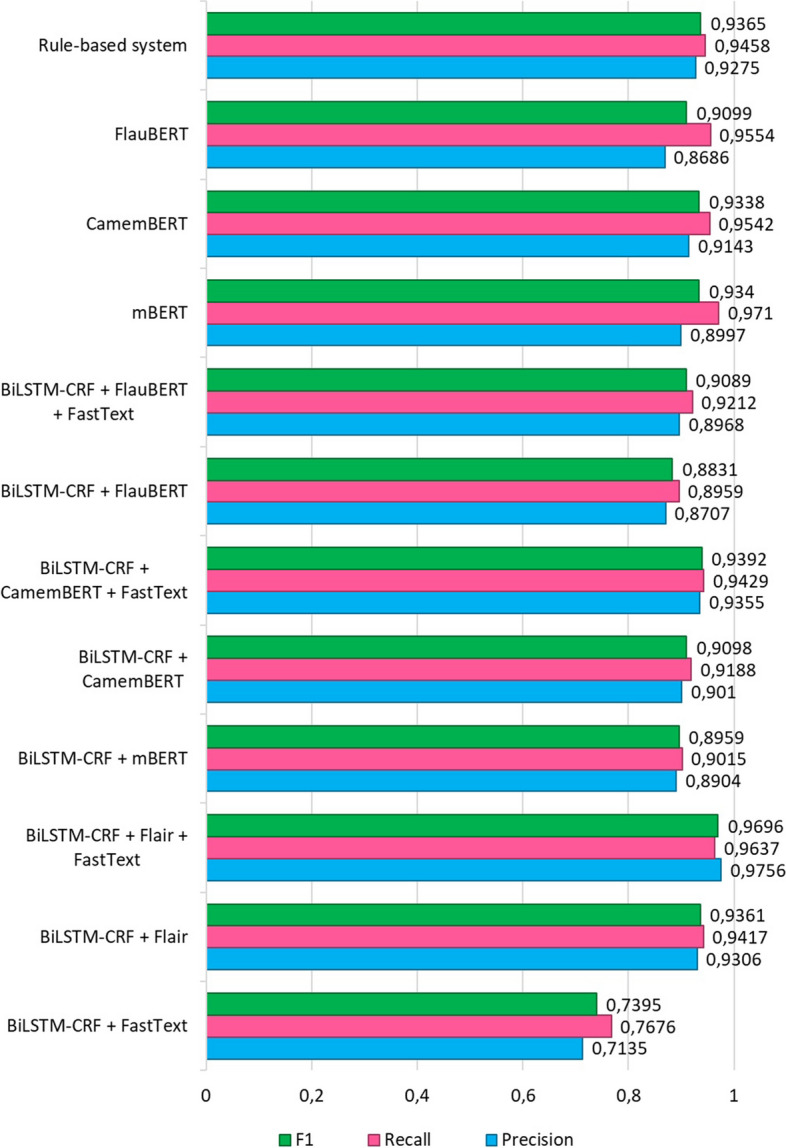
Evaluation results of all tested models

The microaveraged F1 scores for all examined models and embeddings on the manually annotated test dataset are illustrated in Fig. 5. The figure provides a comprehensive comparative analysis of performance metrics—Precision, Recall, and F1 score—specifically focusing on micro-average results across various models evaluated on the manually annotated dataset. The assessed models encompass a diverse range of approaches, including the utilization of Bi-LSTM-CRF with various embeddings (Flair, FastText, BERT, Camembert, and FlauBERT…), fine-tuned language models (BERT, Camembert, and Flaubert), and a rule-based system.

The results of our study indicate that the best-performing model in terms of F1 score is "BiLSTM-CRF + FastText + Flair" with a significant F1 score of 0.9696. This model combines both FastText and Flair embeddings and outperforms the other models. The rule-based system achieves interesting results, with an F1 score of 0.9365. However, some deep learning-based models outperform it, indicating the advantage of pretrained embeddings and neural network architecture (BI-LSTM + CRF) for the NER task.

mBERT and CamemBERT perform well with F1 scores of 0.9340 and 0.9338, respectively, demonstrating the effectiveness of transformer-based language models for named entity recognition. The combination of embeddings (FastText + Flair) generally improves model performance, as shown by the higher F1 scores of the "BiLSTM-CRF + FastText + Flair" model.

The results suggest that combining different types of embeddings and using deep learning models can significantly improve the performance of named entity recognition systems, outperforming rule-based systems for this particular de-identification dataset.

## Performance results per entity class

To further analyze the results, we examined the performance per entity class of the best model and compared it with the rule-based approach. The Detailed Results Obtained by the Best Model Bi-LSTM + CRF with Stacked FastText + FLAIR Embedding are presented in Table 8.

**Table 8:** Detailed results obtained by the best model

Class	Precision	Recall	F1-score	Support
DATE	0.9922	0.9745	0.9832	2078
DOCTOR	0.9283	0.9229	0.9256	1206
VILLE	0.9987	0.9921	0.9954	764
PHONE	0.9832	0.9670	0.9750	545
PATIENT	0.9726	0.9745	0.9736	510
ZIP	1.0000	0.9727	0.9862	293
STR	0.9520	0.9316	0.9414	234
EMAIL	0.9896	0.9896	0.9896	96
Micro avg	0.9756	0.9637	0.9696	5726
Macro avg	0.9771	0.9637	0.9713	5726
Weighted avg	0.9757	0.9637	0.9696	5726

The detailed evaluation results obtained by the rule-based system are presented in Table 9.

**Table 9:** Detailed results obtained by the Rule-based system

Class	Precision	Recall	F1-Score	Support
DATE	0.9927	0.9972	0.9950	2178
DOCTOR	0.9535	0.9196	0.9362	1293
VILLE	0.9713	0.9837	0.9775	860
PHONE	0.9967	0.9869	0.9918	612
PATIENT	0.5530	0.8375	0.6661	480
ZIP	0.9968	0.9231	0.9585	338
STR	0.9105	0.6431	0.7538	269
EMAIL	0.9135	1.0000	0.9548	95
micro avg	0.9275	0.9458	0.9365	6125
macro avg	0.9110	0.9114	0.9042	6125
weighted avg	0.9427	0.9458	0.9408	6125

The performance of each class for the best model shows that the model performs exceptionally well for classes such as "DATE", "CITY", "ZIP" and "EMAIL" with F1 scores of 0.9832, 0.9954, 0.9862 and 0.9896, respectively. For other classes, such as "DOCTOR", "PHONE", "PATIENT" and "STR", the model achieves good F1 scores ranging from 0.9256 to 0.9414.

The rule-based system performs very well for some classes, such as "DATE", "PHONE", "ZIP" and "EMAIL", with F1 scores of 0.9950, 0.9918, 0.9585 and 0.9548, respectively. However, the rule-based system's performance was weaker for "PATIENT" and "STR", with F1 scores of 0.6661 and 0.7538, respectively, indicating that there is a need for improvement.

In conclusion, the best-performing model is "BiLSTM-CRF + FastText + Flair," which combines both FastText and Flair embeddings. It outperforms the other models and achieves an impressive microaveraged F1 score of 96.96%. The rule-based system shows competitive results for some classes but lacks consistency across all classes compared to the best model.

## Performance results of the best model for different documents types

The detailed performance results of our best model ("BiLSTM-CRF + FastText + Flair") for different types of medical documents are available in (Additional file 3). This file provides the performance metrics, specifically micro-average results across various types of medical documents.

The results of this evaluation on different types of medical documents reveal different levels of effectiveness. In particular, the model achieves high scores for the following document types: “Surgical Reports”, with an F1 score of 0.9931. Similarly, in “Other Complementary Examination Report” the model performs exceptionally well, with an F1 score of 0.9902. In “Appointment letters”, the model achieve an F1 score of 0.9825. In addition, the model excelled in extracting PIIs from “hospital stay reports” and “discharge letters” with F1 scores of 0.9926 and 0.9619, respectively.

On the other hand, for some document types, model performance declined, as shown by lower F1 scores. For “Entry letter”, “Patient Letters” and “External documents” the model show an F1 scores of 0.8148, 0.8778 and 0.8108, respectively.

In conclusion, addressing these challenges is essential to improve the overall accuracy of the model and its applicability to a wide range of medical documents. This analysis underlines the importance of continuous monitoring and improvement of the model.

## Discussion

In this study, we proposed a largely automated approach for the de-identification of French clinical reports using automatic annotation of medical text data based on regular rules and knowledge bases. We also proposed a solution for assisted and semiautomatic annotation of EHRs using the Prodigy annotation tool to reduce the cost of creating a reference corpus for the evaluation of our NER models.

It is important to note that our NER models were trained on only one type of document, namely, *hospital discharge summaries*. To study the generalizability of our models, we evaluated their performance on different types of clinical documents, such as *Administrative Records*, *Emergency Room Passes*, *Appointment Notices,* and *Discharge Letters*. The results indicate a high level of agreement among the annotators, confirming the quality and reliability of the annotations made on this set of documents and providing a solid foundation for the evaluation of our NER models.

Our experimental results showed that the proposed approach performed well in extracting PII from clinical notes in unstructured text, with an F1 score of 96.96% on average for the eight entity types considered. Our results also showed that our best deep learning-based model outperformed rule-based systems in terms of F1 score (93.65%). Additionally, the results of this evaluation showed that our models were able to achieve high performance on different types of clinical documents, strengthening their robustness and applicability in the healthcare domain.

Neural networks can incorporate sensitive information, particularly that designed for de-identification tasks. Hence, it is currently difficult to share such models outside the institution that owns the data used to train the model. One advantage of our approach is that it is fully automated, and the whole pipeline can be shared and used by anyone who wants to apply the process to its own data, with little adaptation.

## Comparison to the state-of-the-art

The heterogeneity of electronic health records (EHRs) can make the de-identification process much more complex. In this work, we address this challenge by processing more than 58 document types. Furthermore, while previous work on French clinical de-identification annotates their corpus manually [4, 9], our approach uses distant supervision, which reduces both the cost and time required for annotation.

In addition, we present a comparative study of several NER architectures for the French de-identification task. Our results confirm the effectiveness of the stacked Flair and FastText embeddings combined with the Bi-LSTM + CRF architecture for extracting personal identifiable information from clinical text. Moreover, we extend our comparative study to the de-identification of Italian medical records [6]. The results obtained in their study confirm that the best-performing model for this task also uses the combination of Flair and FastText embeddings, combined with the Bi-LSTM + CRF architecture. This finding demonstrates the robustness of this approach for extracting personally identifiable information.

Although the extracted PII (personally identifiable information) is not quite the same in other French de-identification systems, we can still compare common PII types. This analysis shows that our approach yields similar results to existing French de-identification tools who used manually annotated training bases [9], which demonstrates the effectiveness of our method.

## Limitations of our study

Our study demonstrated the feasibility and effectiveness of using distant supervised learning for the task of deidentifying clinical data, which can help overcome the challenge of limited annotated data in the medical domain.

However, there are some limitations. First, the clinical documents used in our study were retrieved from different sources, and the quality of the reports can vary. Certain medical reports are generally long and poorly formatted documents. The preprocessing of the data is therefore crucial, especially the sentence splitting step. In fact, in poorly formatted documents, sentences can be incomplete or improperly split, which can affect the quality of the annotations and the ability of the model to understand the context in which an entity is mentioned. This difficulty can be reflected in the quality of the de-identification results and the overall performance of the model. Data preprocessing is also crucial for model deployment to ensure that data have the same structure when given to the model for new predictions on unknown examples.

Second, automatic labels are sometimes noisy, which can cause the model to learn incorrect contexts. We would like to improve the model with active learning steps to correct annotations that are incorrect or not detected during the initial training step [59].

The final limitation is to train the model on a corpus from a single hospital, which may result in a lack of generalization. Next, we consider collaborative training across different hospitals by developing a federated method to solve our clinical de-identification problem in a distributed scenario [16].

## Conclusion

In conclusion, our study provides a promising approach for the de-identification of French clinical reports, which can facilitate the reuse of electronic health records for secondary purposes such as clinical research. Our study also highlights the importance of using advanced NLP techniques such as deep learning for effective de-identification, as well as the need for innovative solutions such as distant supervision to overcome the challenge of limited annotated data in the medical domain.

Our work demonstrates that despite the level of noise in the automatically annotated dataset, the trained NER model using the stacked Flair and FastText embeddings combined with the Bi-LSTM + CRF achieves good performance. This is due to the large size of the automatically annotated dataset.

Future work could further explore the scalability and generalization of the proposed approach and investigate its applicability in a collaborative study among multiple medical institutions.

### Supplementary Information

**Additional file 1.** The details of the distribution of document types in the sample. The distribution of document types within the sample is provided, with document categories and corresponding codes. The most common document types include various questionnaires related to medical specialties, such as anesthesia, prescription, physiotherapy, as well as different types of medical records. In addition, there are documents labeled as "Full Document" and a variety of other types of medical questionnaires, each with varying frequency in the dataset.

**Additional file 2. **The detailed annotator concordances by type of document. This file presents annotator agreements for different types of medical documents. The first column contains codes or labels for document categories. The second column describes the document types. The third column contains concordance values, which represent the level of agreement between annotators for each document type. Values range from around 0.509 to 1, with higher values indicating stronger agreement. The data appear to reflect the consistency of annotations between different annotators for various categories of medical documents.

**Additional file 3.** Detailed performance results of the best model ("BiLSTM-CRF + FastText + Flair,") for different types of medical documents. This file provides a detailed overview of performance metrics, specifically micro-average results, for our best model "BiLSTM-CRF + FastText + Flair" across various types of medical documents. The first column ”CODE*_LABEL*” contain labels identifying document categories, while the subsequent columns present precision, recall, and F1-score values. Additionally, the 'Support' column indicates the number of Entities for each document type. These Results are crucial for assessing the effectiveness of our NER model in precisely extracting Personal identifiable information (PPIs) across different medical document categories.

## Data Availability

The data that support the findings of this study are not publicly available to preserve individuals’ privacy under the European General Data Protection Regulation.

## Abbreviations

EHR: Electronic health record
NER: Named entity recognition
CDW: Clinical data warehouse
GDPR: General Data Protection Regulation
CNIL: Commission Nationale de l’Informatique et des Libertés
NLP: Natural language processing
ANN: Artificial neural network
CRF: Conditional random field
Bi-LSTM: Bi-directional long short-term memory
SVM: Support vector machine
GloVe: Global vectors for word representation
BERT: Bidirectional encoder representations from transformers
BAN: Base Adresse Nationale
DRG: Diagnosis-related groups
OMOP: Observational Medical Outcome Partnership
APHP: Assistance Publique des Hôpitaux de Paris
PII: Personal identifiable information
POS: Part-of-speech

## Footnotes

1 https://www.cnil.fr/fr/lanonymisation-de-donnees-personnelles

2 https://ec.europa.eu/info/law/law-topic/data-protection/data-protection-eu_en

3 https://ohdsi.github.io/CommonDataModel/

4 https://www.nltk.org/

5 https://huggingface.co/models
