# Supplementary material for: Automatic de-identification of French electronic health records: a cost-effective approach exploiting distant supervision and deep learning models
Source: BMC Med Inform Decis Mak. 2024 Feb 16;24:54. doi: 10.1186/s12911-024-02422-5 (PMC10870625; doi:10.1186/s12911-024-02422-5)
Supplement: Supplementary file 1 — Additional file 1. The details of the distribution of document types in the sample. The distribution of document types within the sample is provided, with document categories and corresponding codes. The most common document types include various questionnaires related to medical specialties, such as anesthesia, prescription, physiotherapy, as well as different types of medical records. In addition, there are documents labeled as "Full Document" and a variety of other types of medical questionnaires, each with varying frequency in the dataset. [file 12911_2024_2422_MOESM1_ESM.docx]

## Additional file 1

- Title of data: The details of the distribution of document types in the sample.
- Description of data: The distribution of document types within the sample is provided, with document categories and corresponding codes. The most common document types include various questionnaires related to medical specialties, such as anesthesia, prescription, physiotherapy, as well as different types of medical records. In addition, there are documents labeled as "Full Document" and a variety of other types of medical questionnaires, each with varying frequency in the dataset.

| **CATEGORY** | **CODE_LABEL** | **N** |
| --- | --- | --- |
| FORM_CDA | Questionnaire: 01.13-Anesthesia File | 35 |
| FORM_CDA | Questionnaire: Prescription/Realization of Medical Care | 26 |
| FORM_CDA | Questionnaire: 9.3-Physiotherapy | 19 |
| FORM_CDA | Questionnaire: Exam Request Forms | 13 |
| FORM_CDA | Questionnaire: 01.02-Common Medical Data | 12 |
| FORM_CDA | Full Document | 11 |
| FORM_CDA | Questionnaire: 9.1-Dietetics | 10 |
| FORM_CDA | Questionnaire: 01.15B-Gynecology File | 7 |
| FORM_CDA | Questionnaire: 20-Programming | 7 |
| FORM_CDA | 10.2-Psychologist | 6 |
| FORM_CDA | Questionnaire: 01.15A-Common Gynecology-Obstetrics File | 6 |
| FORM_CDA | Questionnaire: 01.27-Infectious Diseases File | 6 |
| FORM_CDA | Questionnaire: 01.34-Ortho-Trauma File | 6 |
| FORM_CDA | Questionnaire: Prescription Regimen | 6 |
| FORM_CDA | Questionnaire: 01.15C-Pregnancy Follow-up File | 5 |
| FORM_CDA | 01.35B-USDSS File | 4 |
| FORM_CDA | 10.1-Social | 4 |
| FORM_CDA | 10.1-Social Activity | 4 |
| FORM_CDA | Questionnaire: 01.04A-Neonatology File | 4 |
| FORM_CDA | Questionnaire: 01.14-Pediatric Surgery File | 4 |
| FORM_CDA | Questionnaire: 01.20-SCHBD File | 4 |
| FORM_CDA | Questionnaire: 01.21-Neurology File | 4 |
| FORM_CDA | Questionnaire: 01.22-Neurosurgery File | 4 |
| FORM_CDA | Questionnaire: 01.30-Cardiology File | 4 |
| FORM_CDA | 9.2-Occupational Therapy | 3 |
| FORM_CDA | Questionnaire: 01.12-Plastic Surgery File | 3 |
| FORM_CDA | Questionnaire: 01.15D-Birth Room File | 3 |
| FORM_CDA | Questionnaire: 01.17-ENT/Cranio-Maxillo-Facial File | 3 |
| FORM_CDA | Questionnaire: 01.18-CTCV File | 3 |
| FORM_CDA | 9.7-Pedicure-Podiatry | 2 |
| FORM_CDA | Questionnaire: 01.05B-MPRE File | 2 |
| FORM_CDA | Questionnaire: 01.07A-Geriatrics File | 2 |
| FORM_CDA | Questionnaire: 01.10-Internal Medicine File | 2 |
| FORM_CDA | Questionnaire: 01.15F-Prenatal Diagnosis File | 2 |
| FORM_CDA | Questionnaire: 01.15G-Contrace | 2 |
| FORM_CDA | Questionnaire: 01.19-Urology File | 2 |
| FORM_CDA | 01.38-Adult Hematology File | 1 |
| FORM_CDA | 01.39-Post-Emergency File | 1 |
| FORM_CDA | 9.4-Speech Therapy | 1 |
| FORM_CDA | 9.5-Orthopedic Prosthetics | 1 |
| FORM_CDA | Questionnaire: 01.03-Pneumology File | 1 |
| FORM_CDA | Questionnaire: 01.04B-Pediatrics File | 1 |
| FORM_CDA | Questionnaire: 01.15E-Postpartum File | 1 |
