# Supplementary material for: Automatic de-identification of French electronic health records: a cost-effective approach exploiting distant supervision and deep learning models
Source: BMC Med Inform Decis Mak. 2024 Feb 16;24:54. doi: 10.1186/s12911-024-02422-5 (PMC10870625; doi:10.1186/s12911-024-02422-5)
Supplement: Supplementary file 2 — Additional file 2. The detailed annotator concordances by type of document. This file presents annotator agreements for different types of medical documents. The first column contains codes or labels for document categories. The second column describes the document types. The third column contains concordance values, which represent the level of agreement between annotators for each document type. Values range from around 0.509 to 1, with higher values indicating stronger agreement. The data appear to reflect the consistency of annotations between different annotators for various categories of medical documents. [file 12911_2024_2422_MOESM2_ESM.docx]

## Additional file 2

- Title of data: The detailed annotator concordances by type of document.
- Description of data: This file presents annotator agreements for different types of medical documents. The first column contains codes or labels for document categories. The second column describes the document types. The third column contains concordance values, which represent the level of agreement between annotators for each document type. Values range from around 0.509 to 1, with higher values indicating stronger agreement. The data appear to reflect the consistency of annotations between different annotators for various categories of medical documents.

| **CODE** | **CODE_LABEL** | **Fleiss** |
| --- | --- | --- |
| DXCARE:266 | 07.1-Emergency admission | 1 (1,1) |
| DXCARE:282 | 04.4-Surgical reports | 1 (1,1) |
| DXCARE:403 | ADMINISTRATIVE | 1 (1,1) |
| DXCARE:DXCARE:LP | 17-Letters to the patient-235 | 1 (1,1) |
| LN:34874-8 | Surgical reports | 0.983 (0.966,1) |
| DXCARE:DXCARE:DIVERS_AUTRES | Miscellaneous reports-226 | 0.973 (0.942,1) |
| DXCARE:DXCARE:LLS | 04.2-Discharge Letters-239 | 0.96 (0.943,0.977) |
| LN:68661-8 | Clinical genetics consultation report | 0.96 (0.93,0.989) |
| SILLAGE:FA | Administrative sheet | 0.957 (0.934,0.981) |
| SILLAGE:COURDIV | Miscellaneous letters | 0.951 (0.915,0.987) |
| EHOP:CR_FONDAMENTUM | FONDAMENTUM report | 0.95 (0.919,0.981) |
| DXCARE:385 | 09.1-Dietary documents | 0.95 (0.911,0.99) |
| SILLAGE:CRE | Endoscopy report | 0.949 (0.926,0.972) |
| DXCARE:377 | 17-Letters to the patient | 0.941 (0.908,0.974) |
| SILLAGE:COURRIER | External correspondence | 0.923 (0.886,0.96) |
| LN:34112-3 | Hospital report (stay) | 0.921 (0.867,0.976) |
| SILLAGE:CR | Report | 0.919 (0.882,0.956) |
| EHOP:CR_ECHO | Ultrasound report (VIEWPOINT) | 0.916 (0.851,0.98) |
| SILLAGE:ETIQ | Label | 0.901 (0.767,1) |
| LN:11490-0 | Discharge letter | 0.855 (0.807,0.904) |
| DXCARE:284 | 04.7-Other complementary exams | 0.852 (0.562,1) |
| SILLAGE:ATT | Certificate | 0.85 (0.732,0.968) |
| SILLAGE:CERTIF | Medical certificate | 0.845 (0.785,0.904) |
| SILLAGE:CONVOC | Appointment letter | 0.797 (0.715,0.88) |
| SILLAGE:ORDON | Prescription | 0.785 (0.664,0.905) |
| LN:15508-5 | Birth report | 0.778 (0.71,0.845) |
| SILLAGE:LACC | Accompanying letter | 0.509 (0.108,0.909) |
