# Supplementary material for: Automatic de-identification of French electronic health records: a cost-effective approach exploiting distant supervision and deep learning models
Source: BMC Med Inform Decis Mak. 2024 Feb 16;24:54. doi: 10.1186/s12911-024-02422-5 (PMC10870625; doi:10.1186/s12911-024-02422-5)
Supplement: Supplementary file 3 — Additional file 3. Detailed performance results of the best model ("BiLSTM-CRF + FastText + Flair,") for different types of medical documents. This file provides a detailed overview of performance metrics, specifically micro-average results, for our best model "BiLSTM-CRF + FastText + Flair" across various types of medical documents. The first column ”CODE_LABEL” contain labels identifying document categories, while the subsequent columns present precision, recall, and F1-score values. Additionally, the 'Support' column indicates the number of Entities for each document type. These Results are crucial for assessing the effectiveness of our NER model in precisely extracting Personal identifiable information (PPIs) across different medical document categories. [file 12911_2024_2422_MOESM3_ESM.docx]

## Additional file 3

- Title of data: Detailed performance results of the best model ("BiLSTM-CRF + FastText + Flair,") for different types of medical documents.
- Description of data: This file provides a detailed overview of performance metrics, specifically micro-average results, for our best model "BiLSTM-CRF + FastText + Flair" across various types of medical documents. The first column ”CODE*_LABEL*” contain labels identifying document categories, while the subsequent columns present precision, recall, and F1-score values. Additionally, the 'Support' column indicates the number of Entities for each document type. These Results are crucial for assessing the effectiveness of our NER model in precisely extracting Personal identifiable information (PPIs) across different medical document categories.

| **Document Types** | **Micro Avg** | | | |
| --- | --- | --- | --- | --- |
| **CODE_LABEL** | **Precision** | **Recall** | **F1-score** | **Support** |
| 10-Doc.divers (Edition) | 0.9887 | 0.9107 | 0.9481 | 672 |
| 04.2-Discharge Letters-239 | 0.9608 | 0.9630 | 0.9619 | 433 |
| Hospital report (stay) | 0.9911 | 0.9940 | 0.9926 | 336 |
| Clinical genetics consultation report | 0.9654 | 0.9767 | 0.9710 | 257 |
| Medical certificate | 0.9637 | 0.9560 | 0.9598 | 250 |
| Miscellaneous letters | 0.9746 | 0.9583 | 0.9664 | 240 |
| FONDAMENTUM report | 0.9712 | 0.9758 | 0.9735 | 207 |
| Consultations or visit reports | 0.9585 | 0.9635 | 0.9610 | 192 |
| Ultrasound report (VIEWPOINT) | 0.9894 | 0.9738 | 0.9815 | 191 |
| External correspondence | 0.9670 | 0.9617 | 0.9644 | 183 |
| Miscellaneous reports-226 | 0.9779 | 0.9725 | 0.9752 | 182 |
| Administrative sheet | 0.9486 | 0.9540 | 0.9513 | 174 |
| Discharge letter | 0.9818 | 0.9818 | 0.9818 | 165 |
| Certificate | 0.9758 | 0.9817 | 0.9787 | 164 |
| 17-Letters to the patient-235 | 0.9615 | 0.9554 | 0.9585 | 157 |
| Patient letter | 0.9801 | 0.9673 | 0.9737 | 153 |
| Birth report | 0.9797 | 0.9667 | 0.9732 | 150 |
| Surgical reports | 0.9931 | 0.9931 | 0.9931 | 144 |
| Report | 0.9640 | 0.9306 | 0.9470 | 144 |
| Medical imaging report | 0.9569 | 0.9652 | 0.9610 | 115 |
| Appointment letter | 0.9825 | 0.9825 | 0.9825 | 114 |
| Report of other examinations compl-223 | 1.0000 | 0.9806 | 0.9902 | 103 |
| Accompanying letter | 0.9798 | 0.9798 | 0.9798 | 99 |
| 17-Letters to the patient | 0.8778 | 0.8778 | 0.8778 | 90 |
| 06.1-Prescriptions-149 | 0.9667 | 0.9667 | 0.9667 | 90 |
| Endoscopy report | 0.9634 | 0.9753 | 0.9693 | 81 |
| Label | 0.9825 | 0.9333 | 0.9573 | 60 |
| Prescription | 0.9828 | 0.9828 | 0.9828 | 58 |
| 04.6-Vigilance report | 0.9778 | 0.9778 | 0.9778 | 45 |
| 04.3-Anesthesia report-220 | 0.8667 | 0.8966 | 0.8814 | 29 |
| 04.8-CR divers autres | 0.9615 | 0.9615 | 0.9615 | 26 |
| Pathological anatomy and cytology report | 0.9565 | 0.8800 | 0.9167 | 25 |
| 04.1-Consultations report | 0.9583 | 0.9200 | 0.9388 | 25 |
| 11.1-Consentements-200 | 0.9565 | 0.9565 | 0.9565 | 23 |
| 06.2-Certificates | 0.8750 | 0.9130 | 0.8936 | 23 |
| Cover letters | 0.8947 | 0.8947 | 0.8947 | 19 |
| 05.1-External documents | 0.7895 | 0.8333 | 0.8108 | 18 |
| Biology test results | 1.0000 | 1.0000 | 1.0000 | 17 |
| 04.4-Surgical reports | 0.9412 | 1.0000 | 0.9697 | 16 |
| Entry letter | 0.8462 | 0.7857 | 0.8148 | 14 |
| 06.1-Prescriptions | 0.9231 | 0.9231 | 0.9231 | 13 |
| 11.1-Consent (editing) | 1.0000 | 0.9231 | 0.9600 | 13 |
